# Gluten as a Food

**Published:** 1891-04

**Authors:** C. P. Pengra

**Affiliations:** Professor of Materia Medica and Botany, Massachusetts College of Pharmacy


### GLUTEN AS A FOOD.

BY C. P. PENGRA, M. D.,
Professor of Materia Medica and Botany, Massachusetts College of Pharmacy.

How little we realize the importance of the foods of our day! Count them and we find that we really have but one kind. Man lives on the vegetable kingdom. True enough, we eat, digest, and assimilate beef, pork, mutton, eggs, and a number more animal *structures*. But are they anything more than modified form of vegetable life? Could any of them exist without the latter? The word structures has been used simply because it expresses the fundamental idea that the animal, man, dependent and living upon the vegetable, is nothing more than a rearrangement of the products of vegetable life. Yes, he modifies them, but he receives, and is glad to accept, and can also live upon the direct products of the plant.

Therefore our inventory of our stock of foods brings us to the products of the soil alone, and we find that our actual supply of food is very limited. In fact, the problem of economic and scientific ages has been and is: "Where is the future food to come from?" Already it has been estimated that a natural soil will inevitably become exhausted in 250 years. Need we follow this line of thought further to lead us to the fact that, if the soil supplies all our wants, it probably produces our necessities? If it produces the necessities of our physiological life, does it not likewise provide for our pathological condition? Admitting this, does it not follow that different products have different purposes, and that in special modifications of the animal system special products of the vegetable are in demand?

Accordingly, it seems reasonable to presume that, for its purpose, the purer the product of the soil the more applicable, useful and direct must be its action. These points have been advanced not only to call attention to the inestimable value of every true food in nature, but also to the idea that as there must be a purpose and place in the animal economy for each and every food, so also must there be demand for the individual constituents of these foods.

The leading physiologists and physicians of to-day are clamoring, not for medicines or new chemical combinations, but for nature, dietetics and proper food. Knowing as we do the importance of this subject, we welcome any addition to our bill of fare that brings evidence of its characteristics and value.

For centuries it has been known that man could live happily upon cereals alone, and it required but little thought to suspect that these very cereals, grain or flour, contained something that substituted the flesh diet of others.

In course of time chemistry developed technically what theory and reason had long supposed, that man obtained from cereals more or less of two kinds of food—a non-nitrogenous (also called starchy, or carbohydrate), and a nitrogenous (meaty or albuminous).

Later—in fact only about forty years ago—we were told that one of the greatest constituents of our vegetable food was gluten. Analysis showed that nature's store of this substance represented from 12 to even 20 per cent. of wheat, 12.6 of oats, 7 of barley, 6 of rice—in fact, that gluten, or some similar nitrogen equivalent, as legumin, vegetable fibrin, etc., is liberally distributed throughout our vegetable diet.

The physiologists promptly applied this discovery, and we were soon made aware that gluten was one of nature's best means of supplying to man the very elements and effects that he sought for and received from the albuminoids, or meats.

They tell us that it is the vegetable food that furnishes stimulation as well as heat, force and energy to the system. Furthermore, as deductions from these principles, they prove to us that this gluten, the nitrogenous food of the vegetable world, must inevitably be one of the greatest foods that are the fuel for all motion, as also chemical action in animal bodies. It is unnecessary to specify proofs of the necessity of nitrogenous food.

The facts that every contraction of a muscle, beat of heart, expansion of lung, secretion and function of digestive fluids, conductivity of nerves, the processes of inflammation, yes, the very vitality of every part of our living bodies, all require and use nitrogen—these are sufficient proofs of the value and need of the best and purest combination of this food element that nature can produce.

As stated above, these properties and values have been greatly accredited to gluten for many years. "The gluten of wheat," "the gluten of oats," "of corn," etc., have become familiar expressions. Likewise the uses of gluten have been specified and its successful applications as to food have long since been pointed out.

No argument that we have seen has failed to direct attention to the fact that its greatest value for its purposes—"food for infants," "diabetics," "nervous debility," and the like—has depended on its "freedom from starch," the point being that in this condition it offered one of nature's simplest and purest forms of nitrogenous food. But what have been the facts?

One of the leading chemists (Ritthausen) has written: "Gluten is composed of . . . ., . . . ., . . . ., . . . ., *and is 12 to 16 per cent. of starch*"—certainly a strange chemical statement, but it nevertheless is an illustration of the explanation of the unsatisfactory results of many "glutens" of the market.

Naturally the greatest expectation has been in the treatment of diabetic, but even these unfortunates have had to labor under a disadvantage, for recent analyses have shown that there was not a a "diabetic food" in the market that did not contain *above* 30 per cent. of starch—in fact, so much of that Dr. Harrington (chemist, Harvard) suggests that ordinary biscuits would be quite as good for this purpose.

Considering these claims for its value and usefulness we have reason to be thankful that so pure and simple a gluten as *Poluboskos* has been placed upon the market.

We recognize Poluboskos as simply what it is claimed to be, "a pure gluten."

No better proof of its purity could be given than the analysis by Dr. Davenport, which shows that four-tenths of one per cent. of it is starch.

A chemist's word gives us a technical story, and also a basis from which we may work out physiological actions and facts.

The lines of use and application of gluten as a food have long been well established, and the following observations and experiences are corrborative of them. In other words, gluten —Poluboskos—is one of the few instances where practice is the greatest proof of theory.

We have all learned that the composition of our first food, the mother's milk, is largely nitrogenous matter; that the egg from which the young chick is developed contains abundance of this material, but merely a trace of carbohydrates; in fact, the laws of nature provide the beginnings of life with foods that produce muscle and strength rather than fat.

It is well known that prior to the third month of life the saliva does not contain ptyaline, the very essential agent that in later life starts the digestion of starchy foods.

Considering these two great evidences, do we need more proof to convince us that if infants needed a starchy diet it would not have been so decidedly opposed by nature? No; the too frequent blunder of "kind friends" in stuffing starchy concoctions down the helpless infant throat has been sufficiently discovered and abolished by the physicians of our day, and most of them are prepared to interdict all carbohydrate foods, or at least see to it that these constituents be so modified as to correspond to the small amount of lactine that is found in the mother's milk.

These are among the leading reasons that have induced the theory that infants in need should be supplied with a nitrogenous rather than a starchy or even mixed diet. These are facts that have led physicians and mothers to long for something to supply the frequent deficiency of nature.

That nature could relieve this want has been believed, and experience is abundant to prove this to be true.

Such is theory.

But what is practice?

The writer's observation and experience with the Crystal Springs pure gluten food, "Poluboskos," has certainly conformed to the foregoing and all accepted theories on the subject of nitrogenous foods. He has seen infants, weak and apparently exhausted from lack of food, stimulated and almost revivified by its use. Where other foods have been rejected by the stomach, this (although dissolved in the same kind of milk that has previously been rejected) is easily retained.

No word of objection or criticism has come to him, and his experience thus far, and that of other physicians and friends, leads to the belief that in this product we have the nearest approach to a natural food for the waning energies of infants and their many ailments of digestion.

Again, older patients continually report its value and relief in cases of weak stomach, dyspepsia, anorexia, etc. Here again theory is sustained, for, regardless of its renovating or tonic effects, it is exceedingly easy of digestion. Observation has shown that this very ease of digestion has been the cause of its retention where other foods have been vomited. Of the many people we have heard say, "Oh, I can't take milk; I either throw it up, or it makes me bilious," I have not known an exception to the report that they are surprised that they can take so much milk with it and feel so well afterwards.

Certainly this is good proof of the well-established theory that "the gluten of vegetables is one of the most rapidly digestible of our foods," and makes its use in stomach disorders correspond in reason to the results of experience.

The use of nitrogenous diet in diabetes is so familiar that its desirability does not even require a physician's recommendation. It is well known. The people know it, while the sufferers from this disease very early become accustomed to directing their own diet.

What has this been? Almost anything in the market. Even they have almost invariably applied lor "gluten, gluten!" But what have they obtained? Many of them in their ambitious determination, having failed to procure their necessary food in this country, have resorted to importation for many years. And with what result? The best and purest obtainable contained from 12 to 30 per cent. of starch. Therefore it is not strange that these people and their advisers have been glad, as the writer knows, to find that their own country and kind are capable of supplying their demands with a purer gluten than they had ever before known.

The writer realizes the frankness of these strong claims, but he also knows that he is dealing with a natural food that makes no claim of secrecy, and is as free to the reader in all its claims as is the beefsteak of the market; yet, while approving its claims, he would go further and say that, besides its usefulness in infant digestion and diabetic disorders, one of its greatest futures will be found in the treatment of nervous diseases.

The theory for this use is very evident. If a food can furnish energy and stimulate force production in the system, how can it do it but by toning up and strengthening the nerves themselves? What, then, must pure gluten be, if it is not one of nature's best nerve tonics?

To prove this, the writer has used Poluboskos in migraine, insomnia (due to nervous debility), in incontinence, and especially in spermatorrhœa, with results that give evidence that it is a nerve food, and that this nitrogenous, vegetable product has a place in the human economy that is not afforded to anything else within our knowledge.

It is not necessary to enter into a comparison of the various foods of the market, because we know of no other preparation, product, or compound that offers us 91 per cent. of nitrogenous food equivalent.

There seems to be every reason to believe that in Poluboskos we are possessed of one of nature's greatest secrets, and that its future place among desirable foods of the table will be only another practical proof of its necessity in the feeding of diseased vitality.

Manufactured by the Crystal Spring Manufacturing Company, Boston.

---

PNEUMONIA.—

℞ Ammoniæ muriatis, ʒ iij.

Antim. et pot tartrat., gr. ij.

Morphiae sulphat, gr. iij.

Syrupi glycyrhizae, ℥ iv.

M. Sig. A teaspoonful every two hours.

—*Dr. N. S. Davis.*

---

FOR CHRONIC BRONCHITIS.—

℞ Morphine sulph., gr. j fs.
Aquae purae, f ʒ ij.
Syr. asclepiadis tuberose,
Syr. pruniae virginianae, aa f ℥ ij.

Solve et. Sig. Shake and take a teaspoonful every 3 to 6 hours. P. R. N.

